# Effect of vildagliptin, a dipeptidyl peptidase 4 inhibitor, on cardiac hypertrophy induced by chronic beta-adrenergic stimulation in rats

**DOI:** 10.1186/1475-2840-13-43

**Published:** 2014-02-13

**Authors:** Toru Miyoshi, Kazufumi Nakamura, Masashi Yoshida, Daiji Miura, Hiroki Oe, Satoshi Akagi, Hiroki Sugiyama, Kaoru Akazawa, Tomoko Yonezawa, Jun Wada, Hiroshi Ito

**Affiliations:** 1 Department of Cardiovascular Therapeutics, Okayama University Graduate School of Medicine, Dentistry and Pharmaceutical Sciences, Okayama, Japan; 2 Department of Cardiovascular Medicine, Okayama University Graduate School of Medicine, Dentistry and Pharmaceutical Sciences, 2-5-1, Shikata-cho, Okayama 700-8558, Japan; 3 Department of Chronic Kidney Disease and Cardiovascular Disease, Okayama University Graduate School of Medicine, Dentistry and Pharmaceutical Sciences, Okayama, Japan; 4 Division of Basic and Clinical Medicine, agano College of Nursing, Nagano, Japan; 5 Center of Ultrasonic Diagnostics, Okayama University Hospital, Okayama, Japan; 6 Department of Molecular Biology and Biochemistry, Okayama University Graduate School of Medicine, Dentistry and Pharmaceutical Sciences, Okayama, Japan; 7 Department of Medicine and Clinical Science, Okayama University Graduate School of Medicine, Dentistry and Pharmaceutical Sciences, Okayama, Japan

**Keywords:** Cardiac hypertrophy, Dipeptidyl peptidase 4 inhibitor, Inflammation, Left ventricular diastolic dysfunction

## Abstract

**Background:**

Heart failure with left ventricular (LV) hypertrophy is often associated with insulin resistance and inflammation. Recent studies have shown that dipeptidyl peptidase 4 (DPP4) inhibitors improve glucose metabolism and inflammatory status. We therefore evaluated whether vildagliptin, a DPP4 inhibitor, prevents LV hypertrophy and improves diastolic function in isoproterenol-treated rats.

**Methods:**

Male Wistar rats received vehicle (*n* = 20), subcutaneous isoproterenol (2.4 mg/kg/day, *n* = 20) (ISO), subcutaneous isoproterenol (2.4 mg/kg/day + oral vildagliptin (30 mg/kg/day, *n* = 20) (ISO-VL), or vehicle + oral vildagliptin (30 mg/kg/day, *n* = 20) (vehicle-VL) for 7 days.

**Results:**

Blood pressure was similar among the four groups, whereas LV hypertrophy was significantly decreased in the ISO-VL group compared with the ISO group (heart weight/body weight, vehicle: 3.2 ± 0.40, ISO: 4.43 ± 0.39, ISO-VL: 4.14 ± 0.29, vehicle-VL: 3.16 ± 0.16, *p* < 0.05). Cardiac catheterization revealed that vildagliptin lowered the elevated LV end-diastolic pressure observed in the ISO group, but other parameters regarding LV diastolic function such as the decreased minimum dp/dt were not ameliorated in the ISO-VL group. Histological analysis showed that vildagliptin attenuated the increased cardiomyocyte hypertrophy and perivascular fibrosis, but it did not affect angiogenesis in cardiac tissue. In the ISO-VL group, quantitative PCR showed attenuation of increased mRNA expression of tumor necrosis factor-α, interleukin-6, insulin-like growth factor-l, and restoration of decreased mRNA expression of glucose transporter type 4.

**Conclusions:**

Vildagliptin may prevent LV hypertrophy caused by continuous exposure to isoproterenol in rats.

## Background

Heart failure with preserved ejection fraction (HFpEF) is the most common form of heart failure in patients with hypertension. HFpEF is associated with considerable morbidity and mortality, and the risk of adverse outcome increases with the severity of diastolic dysfunction [1]. Treatment with anti-hypertensive drugs may be effective for the prevention of HFpEF; however, anti-hypertensive drugs including angiotensin-converting enzyme inhibitor and angiotensin receptor blocker have failed to show a benefit to mortality or morbidity. Interestingly, patients with HFpEF often have systemic insulin resistance [2]. Recent studies have shown that systemic insulin resistance contributes to dysregulated insulin and metabolic signaling in the heart and the development of diastolic dysfunction [3]. Furthermore, insulin resistance is accompanied with chronic low-grade inflammation [4], leading to the pathogenesis of hypertension and chronic heart failure [5,6]. Accordingly, there has been increased interest in restoring energy metabolism and establishing anti-inflammatory therapies for HFpEF.

Glucagon-like peptide-1 (GLP-1 [7-35]) amide is an incretin hormone secreted mainly by the entero-endocrine cells of the intestine in response to the presence of nutrients [7]. GLP-1 receptors are expressed in rodent and human heart [8] and augment the intake of glucose to myocytes to improve energy metabolism [9]. GLP-1 was also reported to enhance L-type Ca^2+^ current in isolated cardiomyocytes via activation of the cAMP-dependent protein kinase A mechanism [10]. An experimental study showed that a GLP-1 analog had protective effects on high-fat diet–induced insulin resistance [11], inflammation [12], and myocardial infarction [13]. However, the in vivo half-life of GLP-1 is very short (2 to 3 min) because it is degraded by dipeptidyl peptidase 4 (DPP4), and thus DPP4 inhibitors lead to increased GLP-1 levels in the blood and extend the duration of GLP-1 action. Recent experimental studies evaluated the protective effect of DPP4 inhibitors in cardiovascular disease in hypertension [14], heart failure [15], and myocardial infarction [16-18].

Recently, we reported that continuous infusion of isoproterenol (ISO) induces cardiac hypertrophy and diastolic dysfunction in rats [19]. Despite the above-mentioned studies on cardiac function, the effects of DPP4 inhibition on cardiac inflammation and cardiac insulin resistance have not been fully elucidated. Therefore, the purpose of this study was to investigate whether a DPP4 inhibitor, vildagliptin, can prevent left ventricular (LV) hypertrophy and LV diastolic function in isoproterenol-treated rats.

## Methods

### Animals and drug infusion

Male Wistar rats weighing 193–222 g (8 weeks old; CLEA Japan, Inc.) were used as described [19]. Delivery of isoproterenol or vehicle was achieved by subcutaneously implanting an osmotic minipump (Alzet, model 2001; 1.0 μL/h) in the neck under diethyl ether inhalation anesthesia. Rats were divided into four groups and treated for 7 days: vehicle group (subcutaneous pH 4.0 HCl in saline, *n* = 10), ISO group (subcutaneous isoproterenol 2.4 mg/kg/day, *n* = 20), isoproterenol + vildagliptin group (ISO-VL; subcutaneous isoproterenol 2.4 mg/kg/day and oral vildagliptin 30 mg/kg/day, *n* = 20), and vehicle + vildagliptin group (subcutaneous pH 4.0 HCl in saline and oral vildagliptin 30 mg/kg/day, *n* = 20). Vildagliptin was administered by gavage in 0.5% carboxymethyl cellulose sodium. All animal protocols were approved and conducted according to the recommendations of the Okayama University Animal Care and Use Committee.

### Oral glucose tolerance test

After rats were fasted for 18 h, glucose was orally administered (2 g/kg). Blood samples were obtained before and 30, 60, 90, and 120 min after glucose loading. Blood glucose concentrations were measured immediately with a blood glucose monitor (Accu-Check, Roche), and data were quantified by calculating the area under the curve (AUC 0–120 min) using the trapezoidal rule.

### Measurements of plasma active GLP-1, brain natriuretic peptide, and pentosidine

Active (uncleaved, 7–36 amide or 7–37) GLP-1 was detected using the commercially available enzyme-linked immunosorbent assay kit (Millipore) using mouse insulin standards; each sample was analyzed in duplicate. This antibody only detects active GLP-1 (7–36 amide or 7–37 GLP-1) but not cleaved GLP- 1 (9–36 amide or 9–37 GLP-1). Plasma brain natriuretic peptide and pentosidine were measured using an enzyme-linked immunosorbent assay method at SRL Company Ltd. (Tokyo, Japan).

### DPP4 activity measurements

DPP4 activity was determined by the cleavage rate of 7-amino-4-methylcoumarin (AMC) from the synthetic substrate *H*-glycyl-prolyl-AMC (Gly-Pro-AMC; Sigma). Briefly, 5 μl of sample was mixed with 35 μl of assay buffer (25 mM HEPES). After 5-min preincubation at room temperature, the reaction was initiated by the addition of 40 μl of assay buffer containing 0.1 mM substrate Gly-Pro-AMC. After incubation for 20 min, fluorescence was determined using a spectrofluorometer (excitation 380 nm/emission 460 nm). The standard curve of free AMC was generated using 0–50 mM AMC (Sigma). DPP4 activity in plasma was expressed as the amount of cleaved AMC per minute per ml (nmol/min/ml).

### Echocardiography

Seven days after infusion, transthoracic echocardiography was performed using a 10-MHz phased array transducer (Aplio ver. 6.0, Toshiba, Japan) under 2% isoflurane. An electrocardiogram was acquired simultaneously. End diastole was defined as the peak of the R wave, and end systole was defined as the end of the T wave. All animals underwent echocardiographic interrogation while lying in a left recumbent position. Parasternal short-axis and apical long-axis views were obtained. M-mode echocardiography was performed using a parasternal short-axis view at the level of the papillary muscles. LV posterior and interventricular septal diastolic wall thicknesses (PWT and IVST) were measured during diastole (d) and systole (s) as were the LV internal diameters at end diastole (LVDd) and end systole (LVDs). Fractional shortening (FS) was then calculated according to the formula FS = [(LVDd - LVDs)/LVDd] × 100. LV mass (LVM) was calculated according to 1.04 × [(LVDd + PWT + IVST)^3^ – (LVDd) ^3^] [20] and LV mass index (LVMI) was calculated by normalizing LVM for body weight. The apical four-chamber view was used to assess early and late transmitral peak diastolic flow velocities (E and A waves, respectively).

### Hemodynamic measurements

Seven days after infusion, rats were anesthetized, and a micro-tip pressure transducer (Millar Instruments Inc., Houston, TX, USA) was inserted into the right carotid artery. Arterial systolic and diastolic blood pressures were recorded in the aortic arch. The catheter was advanced into the LV cavity. After a 5-min period of stabilization, heart rate, LV systolic pressure (LVSP), LV end-diastolic pressure (LVEDP), and developed LV pressure (dLVP = LVSP - LVEDP) were measured. For indices of contractility and relaxation, the maximal rates of increase and decrease in LVP dp/dt maximum and dp/dt minimum were determined.

### Histology

The LV was fixed with 4% paraformaldehyde in phosphate buffered saline (PBS), embedded in paraffin, and cut into 5-μm-thick sections. Sections were stained with hematoxylin and eosin for morphological analysis and with Masson’s trichrome to detect fibrosis and then examined by light microscopy. In sections stained with Masson’s trichrome, interstitial fibrosis was measured using computer-assisted image analysis, and the percent fibrosis was calculated [21,22]. The widths of 30 individual cardiomyocytes in each group were measured as previously described. To visualize the capillaries in the myocardium, endothelial cells in frozen sections were stained with anti-rat CD31 Ab (BD Pharmingen) at 1:100 dilution. The antibody was visualized with a horseradish peroxidase (HRP)-conjugated goat anti-rat IgG and streptavidin-HRP complex (Vectastain ABC elite kit, Vector). HRP activity was detected with 0.025% diaminobenzidine and 0.03% H_2_O_2_ in PBS. Images were captured with a light microscope, and capillaries were evaluated as the number of vessels per cardiomyocyte.

### Quantitative PCR

Total RNA was extracted from heart tissue using Trizol (Invitrogen, Carlsbad, CA, USA). Total RNA (2 μg) was reverse-transcribed using ReverTra Ace (TOYOBO, Osaka, Japan). Finally, the cDNAs were diluted 5-fold before conventional reverse transcription-PCR (RT-PCR) amplification or 50-fold before quantitative PCR analysis. Quantitative real-time RT-PCR was performed using a LightCycler rapid thermal cycler system (Roche Applied Science) as reported [23]. The PCR primers were as follows (forward primer and reverse primer): tumor necrosis factor-alpha (*Tnfa*), 5′-TGAACTTCGGGGTGATCG-3′ and 5′- GGGCTTGTCACTCGAGTTTT-3′; interleukin-6 (*Il6*), 5′-GCCCTTCAGGAACAGCTATG-3′ and 5′-GCAGTGGCTGTCAACAACA-3′; α-myosin heavy chain (*Myh6*), 5′-CATGCGCATTGAGTTCAAGA-3′ and 5′-TCATCCACGGCCAATTCT-3′; glucose transporter type 4 (*Glut4*), 5′-TTGCAGTGCCTGAGTCTTCTT-3′ and 5′-CCAGTCACTCGCTGCTGA-3′; insulin-like growth factor-l (*Igf1*), 5′-ATGCCCAAGACTCAGAAGGA-3′ and 5′-CGTGGCATTTTCTGTTCCTC-3′; dipeptidylpeptidase 4 (*Dpp4*), 5′-CTCCAGAGGACAACCTTGAC-3′ and 5′-GGACAAGTGTGCTCTTGAGT-3′; glyceraldehyde 3-phosphate dehydrogenase (*Gapdh*), 5′-GGCAAGTTCAATGGCACAGT-3′ and 5′-TGGTGAAGACGCCAGTAGACTC-3′. *Gapdh* served as an internal control. Data were analyzed using the standard curve method.

### Statistical analysis

All data are expressed as the mean ± SE. The two-tailed Student’s *t*-test for two groups and one-way analysis of variance followed by Tukey post-hoc test for more than two groups were used. *p*-values < 0.05 were considered significant.

## Results

### Oral glucose tolerance test and active GLP-1 levels

**Figure 1:**
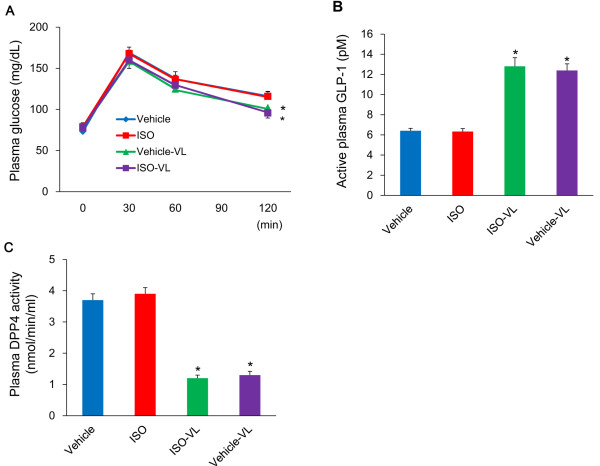
**Effects of vildagliptin on glucose tolerance, active GLP-1 levels, and DPP4 activity in isoproterenol-treated rats.** Plots of blood glucose over time **(A)**, levels of active plasma glucagon-like peptide (GLP)-1 **(B)**, and dipeptidyl peptidase (DPP)-4 activity **(C)** in vehicle, ISO, ISO-VL, and vehicle-VL groups. Data represent the mean ± SE of 10 rats. *p < 0.05 vs. the vehicle and ISO groups.

Oral glucose tolerance and plasma GLP-1 levels in the vehicle, ISO, vehicle-VL, or ISO-VL groups are shown in Figure 1. The glucose levels at 120 minutes after glucose loading were significantly lower in the ISO-VL and vehicle-VL groups than in the vehicle and ISO groups (Figure 1A). AUCs were also significantly smaller in the ISO-VL and vehicle-VL groups than in the vehicle and ISO groups (ISO-VL, 244 ±7 mg; vehicle-VL 243 ± 6 mg min/dl; vehicle, 264 ± 7 mg min/dl: ISO, 265 ± 8 mg min/dl, *p* < 0.05). Active GLP-1 levels at the fasting state were significantly higher in the ISO-VL and vehicle-VL groups than in the vehicle and ISO groups (Figure 1B), and DPP4 activity at the fasting state was significantly lower in the ISO-VL and vehicle-VL groups than in the vehicle and ISO groups (Figure 1C).

### Body and heart weight

Body weight, heart weight, and the heart-to-body weight ratio after 7 days of treatment are shown in Table 1. We observed no difference in body weight among the four groups at baseline or at 7 days post-treatment with vehicle, ISO, vehicle-VL, or ISO-VL. The increases in heart weight and heart-to-body weight ratio observed for the ISO group were significantly suppressed in the ISO-VL group (*p* < 0.05). Vildagliptin alone did not affect heart weight or heart-to-body weight ratio. Therefore, histological, hemodynamic and gene expression analyses were performed only in rats treated with vehicle, ISO, or ISO-VL.

**Table 1:** Effects of vildagliptin on heart weight

	**Vehicle**	**ISO**	**ISO-VL**	**Vehicle-VL**
Number of rats	10	20	20	20
Body weight, g	237 ± 7	235 ± 10	234 ± 11	237 ± 6
Heart weight, g	0.76 ± 0.10	1.04 ± 0.11*	0.97 ± 0.10*#	0.75 ± 0.09
Heart weight/body weight × 10^3^	3.21 ± 0.40	4.43 ± 039*	4.14 ± 0.29*#	3.16 ± 0.16

ISO, isoproterenol; VL, vildagliptin. Values represent the mean ± SE. *p < 0.05 vs. vehicle and vehicle-VL; #*p* < 0.05 vs. ISO.

### Histology

**Figure 2:**
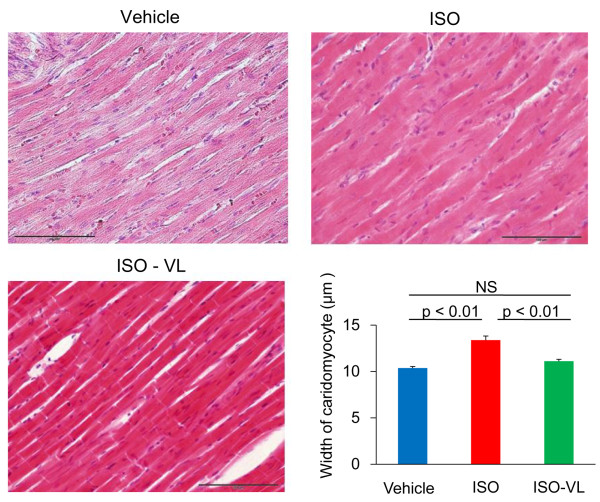
**Effect of vildagliptin on cardiomyocyte size in isoproterenol-treated rats.** Hematoxylin and eosin staining of hearts in the vehicle, ISO, and ISO-VL treatment groups. The widths of 30 individual cardiomyocytes in rat were measured across a line bisecting the nucleus. Scale bar = 100 μm. Bar graph shows the cardiomyocyte width. Values represent the mean ± SE. *n* = 6 each. NS = not significant.

**Figure 3:**
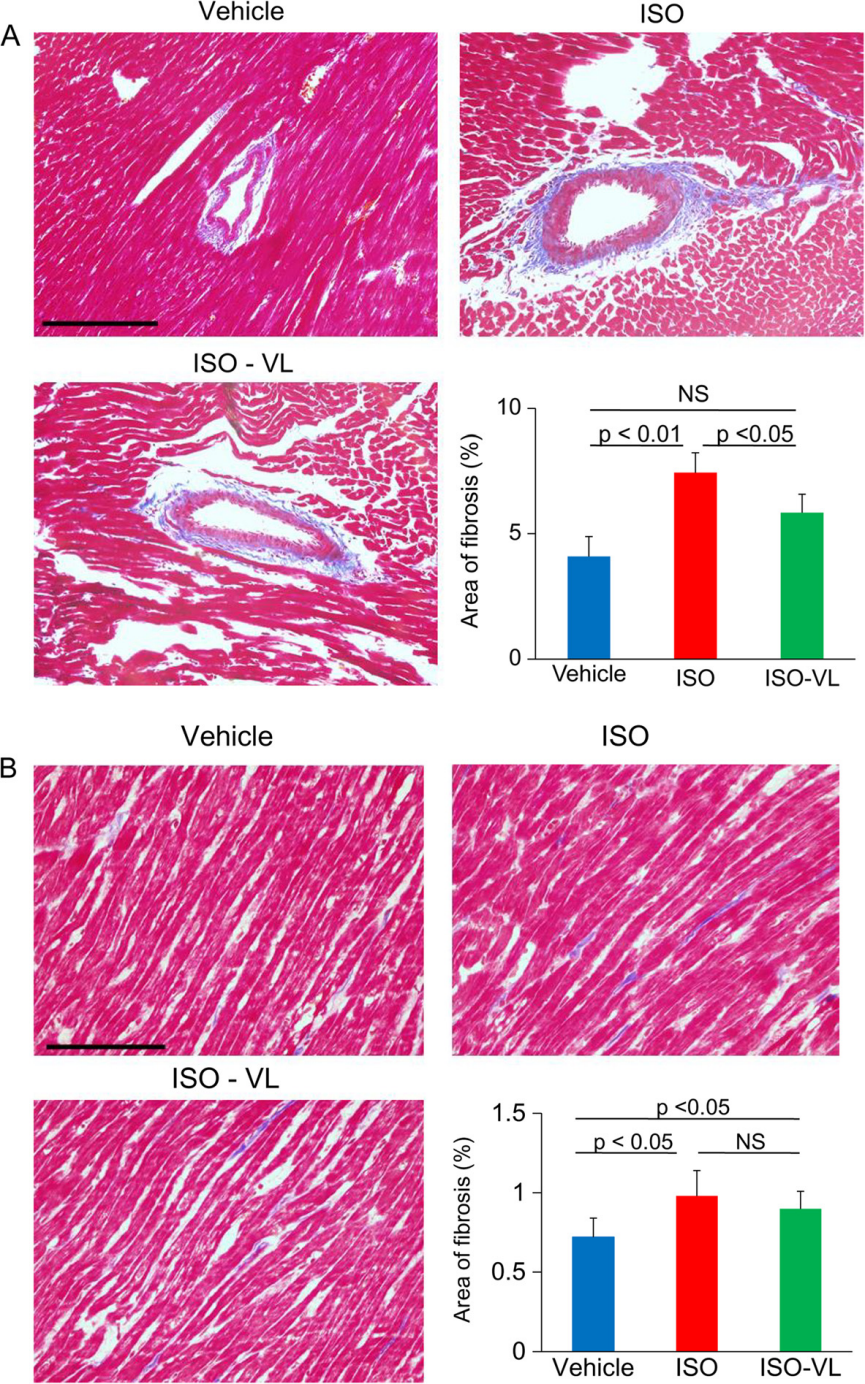
**Effect of vildagliptin on cardiac fibrosis in isoproterenol-treated rats.** Masson’s trichrome staining of hearts in the vehicle, ISO, and ISO-VL treatment groups. **A**: Perivascular fibrosis. **B**: Interstitial fibrosis. Scale bars = 100 μm. Bar graphs show the area of fibrosis (%) in each case. Values represent the mean ± SE. *n* = 6 each. NS, not significant.

**Figure 4:**
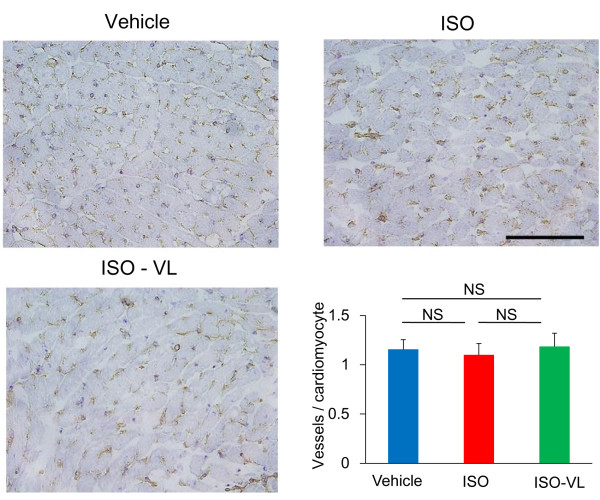
**Effect of vildagliptin on capillary vessels in isoproterenol-treated rat myocardium.** CD31 staining of hearts in the vehicle, ISO, and ISO-VL groups. Scale bar = 100 μm. Bar graph shows the number of vessels per cardiomyocyte. Values represent the mean ± SE. *n* = 6 each. NS, not significant.

Hematoxylin and eosin staining after 7 days of treatment revealed that the increase in cardiomyocyte width in the ISO group was significantly suppressed in the ISO-VL group (cardiomyocyte width: vehicle, 10.4 ± 0.2 μm; ISO, 13.4 ± 0.4 μm; ISO-VL, 11.1 ± 0.2 μm; Figure 2). Masson’s trichrome staining revealed that the area of perivascular fibrosis was significantly suppressed in the ISO-VL group compared with the ISO group (area of fibrosis: vehicle, 4.1 ± 1.8%; ISO, 7.4.5 ± 1.8%; ISO-VL, 5.8 ± 1.6%; Figure 3A), but vildagliptin did not limit the increased area of interstitial fibrosis caused by isoproterenol (area of fibrosis: vehicle, 0.72 ± 0.1%; ISO, 0.98 ± 0.2%; ISO-VL, 0.90 ± 0.1%; Figure 3B). CD31 staining revealed that the number of CD31-positive capillaries/cardiomyocyte did not differ among the three groups (vessels/cardiomyocyte: vehicle, 1.2 ± 0.1%; ISO, 1.1 ± 0.1%; ISO-VL, 1.2 ± 0.1%; Figure 4).

### LV Hemodynamic and echocardiographic data

In non-invasive tail-cuff blood pressure measurements, systolic blood pressure at day 7 was significantly higher in the ISO-VL group than in the vehicle and ISO groups (127 ± 6, 119 ± 5, and 117 ± 5 mmHg, respectively, p < 0.05); however, cardiac catheterization showed no differences in systolic pressure among the three groups. As shown in Table 2, hemodynamic data by cardiac catheterization revealed diastolic dysfunction in ISO-treated rats. ISO-treated rats showed significantly increased maximum dp/dt, depressed minimum dp/dt, and elevated LVEDP compared with vehicle**-**treated rats, but there was no significant difference in LVSP between the three groups. Vildagliptin significantly improved LVEDP in ISO-treated rats, whereas no significant differences were found in maximum dp/dt or minimum dp/dt between the ISO group and the ISO-VL group. Echocardiography showed no differences in LV systolic function among the three groups, and vildagliptin attenuated the increase in LVM and LVMI in ISO-treated hearts (Table 2).

**Table 2:** Effects of vildagliptin on LV hemodynamic and echocardiographic data

	**Vehicle**	**ISO**	**ISO-VL**
***Cardiac catheterization***			
Number of rats	10	10	10
Heart rate (bpm)	404 ± 8	519 ± 7*	491 ± 8* #
LVSP (mmHg)	108 ± 3	113 ± 2	116 ± 2
LVEDP (mmHg)	2.2 ± 1.1	3.4 ± 0.9*	2.5 ± 1.0#
Maximum dp/dt (mmHg/s)	8992 ± 434	16,969 ± 592*	16,189 ± 713*
Minimum dp/dt (mmHg/s)	-8251 ± 531	-9936 ± 459*	-10080 ± 236*
***Echocardiography***			
Number of rats	10	10	10
LVDd (mm)	6.78 ± 0.14	6.71 ± 0.04	6.74 ± 0.08
LVDs (mm)	3.62 ± 0.14	3.29 ± 0.08	3.41 ± 0.07
FS (%)	46.7 ± 2.7	51.0 ± 1.1	50.0 ± 0.9
IVST (mm)	1.34 ± 0.04	1.57 ± 0.23*	1.51 ± 0.03*
PWT (mm)	1.38 ± 0.04	1.62 ± 0.03*	1.56 ± 0.03*
E/A	1.61 ± 0.24	1.57 ± 0.07	1.53 ± 0.05
LVM (mg)	567 ± 16	695 ± 9*	663 ± 11* #
LVMI (mg/g)	2.36 ± 0.06	2.96 ± 0.04*	2.83 ± 0.04* #

ISO, isoproterenol; VL, vildagliptin; LVSP, left ventricular systolic pressure; LVEDP, left ventricular end diastolic pressure; LVDd, left ventricular end-diastolic diameter; LVDs, left ventricular end-systolic diameter; FS, fractional shortening; IVST, interventricular septal thickness; PWT, posterior wall thickness; E, early transmitral peak diastolic flow velocity; A, late transmitral peak diastolic flow velocity; LVM, left ventricular mass; LVMI, left ventricular mass index. Values represent the mean ± SE. *p < 0.05 vs. vehicle; #*p* < 0.05 vs. ISO.

### Cardiac gene expression

**Figure 5:**
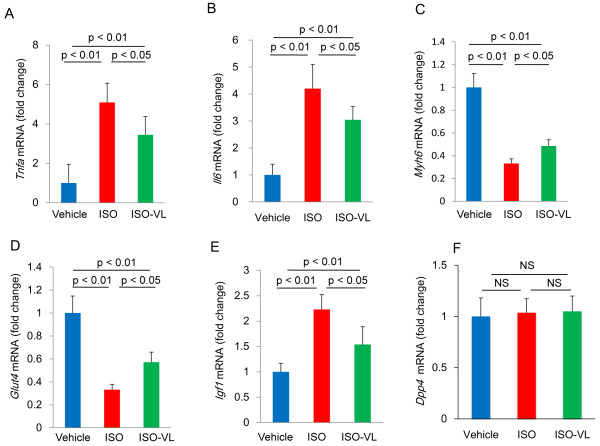
**Cardiac gene expression in isoproterenol-treated rats.** The changes in expression of tumor necrosis factor alpha (*Tnfa*) **(A)**, interleukin-6 (*Il6*) **(B)**, α-myosin heavy chain (*Myh6*) **(C)**, glucose transporter type 4 (*Glut4*) **(D)**, insulin-like growth factor (*Igf1*) **(E)**, and dipeptidyl peptidase-4 (*Dpp4*) **(F)** mRNAs were analyzed using quantitative PCR. Values represent the mean ± SE. *n* = 8 each. NS, not significant.

Figure 5 shows levels of mRNA expression in the heart after treatment. The increased expression of genes that encode inflammatory markers such as Tnfa and Il6 upon ISO treatment was significantly suppressed by vildagliptin (Figure 5A and B). Expression of the gene that encodes Myh6, which is the dominant major histocompatibility complex (MHC) isoform expressed in normal hearts, was significantly decreased in the ISO group (Figure 5C). Vildagliptin significantly limited the decrease in expression of *Myh6* mRNA. The mRNA expression of the gene that encodes Glut4, which mediates glucose uptake, was significantly reduced by isoproterenol treatment. Vildagliptin also significantly limited the decrease in *Glut4* mRNA (Figure 5D). The increased expression of *Igf1* mRNA upon isoproterenol treatment was significantly suppressed by vildagliptin (Figure 5E). The expression of *Dpp4* mRNA was not affected by isoproterenol treatment (Figure 5F).

### Measurements of brain natriuretic peptide and pentosidine

After 7 days of treatment, the increase in brain natriuretic peptide (BNP) level in the ISO group was significantly suppressed in the ISO-VL group (vehicle, 52.2 ± 2.3 pg/ml; ISO, 74.7 ± 6.9 pg/ml; ISO-VL, 62.0 ± 2.3 pg/ml, *p* < 0.05). There was no difference in the plasma levels of pentosidine, which is an advanced glycation end product and is generated by non-enzymatic glycation and oxidation of proteins (vehicle, 9.4 ± 0.51 μg/ml; ISO, 8.8 ± 0.63 μg/ml; ISO-VL, 9.4 ± 0.52 μg/ml).

## Discussion

We demonstrated that short-term treatment with a DPP4 inhibitor, vildagliptin, prevented LV hypertrophy caused by continuous infusion of isoproterenol. These effects were accompanied by the amelioration of expression of genes associated with glucose uptake and inflammation. Although cardiac catheterization showed that vildagliptin did not significantly improve LV diastolic dysfunction in isoproterenol-treated rats, this study indicated that vildagliptin has potential for preventing LV hypertrophy independent of blood pressure.

Regarding the mechanisms underlying the protective effect of vildagliptin in preventing isoproterenol-induced LV hypertrophy in this study, one possible explanation is that vildagliptin may reduce inflammation in the heart. A previous study showed that isoproterenol induces expression of mRNAs that encode myocardial pro-inflammatory cytokines such as TNF-α, IL-6, and IL-1β [24]. Our previous study revealed a direct effect of TNF-α on cardiac hypertrophy in cultured cardiomyocytes [25]. Thus, TNF-α and IL-6 are likely to be important factors in the induction of hypertrophy [26,27]. In our current study, vildagliptin reduced the expression of *Tnfa* and *Il6* mRNAs in myocardial tissue of isoproterenol-treated rats. Similarly, recent studies demonstrated the effect of DPP4 inhibitors on the reduction of pro-inflammatory cytokines in macrophages, visceral adipose tissue, and atherosclerotic plaques [28,29]. Furthermore, vildagliptin suppressed the increase in *Igf1* expression induced by isoproterenol in our rat model. Recent studies showed the involvement of IGF1 in cardiomyocyte hypertrophy [30,31]. Thus, a change in cytokine expression by vildagliptin may contribute to the prevention of LV hypertrophy. Another possibility is that increased active GLP-1 levels by vildagliptin directly influences LV hypertrophy. In several previous experiments in rats, oral vildagliptin was used at a dose of 3 to 60 mg/kg/day [11,32]. We selected the dose of 30 mg/kg/day of vildagliptin in this study and confirmed that this dosage increased the GLP-1 level at the fasting state by 2-fold compared with the control group. A study showed that recombinant GLP-1 infusion for 14 days reduces blood pressure, LV hypertrophy, and LV fibrosis in Dahl salt-sensitive rats [33]. Another group has reported that administration of a GLP-1 analog diminishes cardiac hypertrophy and blood pressure in obese mice exhibiting insulin resistance [34]. In both studies, it was difficult to discriminate the effect of GLP-1 on the protection of LV hypertrophy from its blood pressure–lowering effects. Taken together, these results suggest that the anti-inflammatory effect and suppression of IGF1 by vildagliptin in the heart at least partly counters LV hypertrophy.

In this study, although LVEDP was significantly lower in the ISO-VL group than in the ISO group, other catheter-related parameters such as maximum dp/dt, minimum dp/dt were similar between the two groups. Thus, this study failed to demonstrate that vildagliptin ameliorated LV diastolic function in the ISO-VL group. Energy metabolism, however, switches from fatty acid oxidation to carbohydrate oxidation in hypertrophied hearts [35], and thus the increase in expression of *Glut4* mRNA by vildagliptin may improve glucose uptake in cardiomyocytes and then ameliorate ATP synthesis through carbohydrate oxidation. In fact, treatment with a DPP4 inhibitor, sitagliptin, improves insulin resistance and increases cardiac GLUT4 protein and mRNA abundance in spontaneously hypertensive rats [36]. In hypertrophied hearts, the shift in MHC isoform composition from α- to β-MHC has also been reported [37]. Our finding that expression of cardiac α-MHC (*Myh6*) mRNA decreased in isoproterenol-treated rats is consistent with those previous data. The improvements in energy production have beneficial effects on failing hearts and may upregulate *Myh6* expression. In this study, although insulin sensitivity was not assessed, rats in the ISO and vehicle groups exhibited similar glucose tolerance patterns. Given that vildagliptin lowered the AUCs in both isoproterenol-treated and vehicle-treated rats, vildagliptin may exert favorable effects on insulin signaling in isoproterenol-treated hearts. This study also demonstrated that decreased inflammatory cytokines may contribute to dysregulated cardiac signaling. Other studies have shown that TNF-α causes cardiac insulin resistance by inducing degradation of insulin receptor substrate protein 1, which is critical for cardiac insulin signaling [38,39]. Thus, reduced expression of *Tnfa* mRNA by vildagliptin, as shown in our present study, also partly contributes to the increase in energy production in isoproterenol-treated hearts. On the other hand, histological analyses in this study showed that vildagliptin significantly suppressed perivascular fibrosis in isoproterenol-treated hearts but did not affect angiogenesis. One study showed that vildagliptin reverses angiogenesis in diabetic murine hearts by increasing the activity of stromal cell–derived factor-1α, which is a substrate of DPP4 [40]. The lack of change in angiogenesis in the isoproterenol-infused rat model may explain why diastolic function is not significantly improved.

DPP4 is widely expressed on the surface of endothelial cells and immune cells such as lymphocytes and monocytes [40-42]. DPP4 inhibitors exert their effects by inhibiting enzymatic degradation of GLP-1; however, recent studies reported non-enzymatic functions for DPP4 [43]. For instance, cell-surface DPP4 regulates inflammatory responses in innate immune cells such as monocytes and dendritic cells through the incretin-independent pathway [44,45]. In endothelial cells, DPP4-mediated signaling pathways result in phosphorylation of endothelial nitric oxide synthase [41]. The distinct role of DPP4 in cardiac tissue remains unknown, but further examination will shed light on the novel functions of DPP4 inhibitors in the heart.

Although the effects of DPP4 inhibitors on LV hypertrophy in humans have not been fully elucidated, the moderate blood pressure–lowering effect of DPP4 inhibitor may have favorable effect on LV hypertrophy in this clinical setting [46]. The mechanism of this effect has been attributed to increased diuresis and natriuresis owing to inhibition of sodium reabsorption from the proximal renal tubules [47]. Another mechanism for the blood pressure–lowering effects of DPP4 inhibitors is peripheral vasodilatation and decreased peripheral vascular resistance. Recently, van Poppel et al. showed that vildagliptin improves endothelial function in patients with type 2 diabetes [48]. Moreover, a recent clinical study indicated that endothelial dysfunction is closely associated with HFpEF [49], and thus favorable effects of DPP4 inhibitors on the vascular system suggest a therapeutic potential for preventing LV hypertrophy and HFpEF in humans.

### Limitations

The elevation of the circulating GLP-1 level has a potential role in cardioprotection, but we did not explore the direct effect of GLP-1 on hypertrophy of cardiomyocytes in this study. Further, a wide range of peptides are considered to be substrates of DPP4. Comprehensive analysis of the effect of DPP4 inhibitors on the substrates in hypertrophied hearts will help to identify the underlying mechanisms. Second, our finding indicated the potential role of vildagliptin on the protection, but not regression, of LV hypertrophy. In clinical practice, it would be of interest to see if administration of DPP4 inhibitor has benefits for patients with existing LV hypertrophy. Further study is warranted to address this question. Finally, It is necessary to understand the role of DPP4 itself in cardiac hypertrophy to address the potential role of DPP4 inhibitors as therapeutics.

In conclusion, a DPP4 inhibitor, vildagliptin, prevented LV hypertrophy caused by continuous exposure to isoproterenol in rats. This finding suggests the possibility of using a DPP4 inhibitor to prevent LV hypertrophy in humans.

## Abbreviations

A: Late transmitral peak diastolic flow velocity; DPP4: Dipeptidyl peptidase 4; E: Early transmitral peak diastolic flow velocity; FS: Fractional shortening; GAPDH: Glyceraldehyde 3-phosphate dehydrogenase; GLP-1: Glucagon-like peptide-1; GLUT4: Glucose transporter type 4; HFpEF: Heart failure with preserved ejection fraction; IGF: Insulin-like growth factor-l; IL6: Interleukin-6; ISO: Isoproterenol; LVMI: Left ventricular mass index; LVDs: Left ventricular internal diameters at end systole; LVEDP: Left ventricular end diastolic pressure; LVSP: Left ventricular systolic pressure; IVST: Interventricular septal diastolic wall thicknesses; IVST: Interventricular septal diastolic wall thicknesses; LVDs: Left ventricular internal diameters at end systole; MYH6: α-myosin heavy chain; PWT: Posterior wall thickness; TNFA: Tumor necrosis factor-alpha; VL: Vildagliptin.

## Competing interests

JW is a consultant for Boehringer Ingelheim, receives speaker honoraria from Novartis.

## Authors’ contributions

TM and KN conceived the study, participated in its design and coordination, and drafted the manuscript. MY, DM, HO, SA, HS, KA, and TY made substantial contributions to acquisition of data or analysis. JW and HI were involved in drafting the manuscript or revising it critically for important intellectual content. All authors’ read and approved the final manuscript.
